# Does elevated *p*CO_2_ affect reef octocorals?

**DOI:** 10.1002/ece3.351

**Published:** 2012-11-26

**Authors:** Yasmin Gabay, Yehuda Benayahu, Maoz Fine

**Affiliations:** 1 Department of Zoology, Tel-Aviv UniversityTel-Aviv, 69978, Israel; 2 The Interuniversity Institute for Marine ScienceP.O. Box 469, Eilat, 88013, Israel; 3 The Mina and Everard Goodman Faculty of Life Sciences, Bar-Ilan UniversityRamat-Gan, 52900, Israel

**Keywords:** Climate change, ocean acidification, octocorals, Red Sea

## Abstract

Increasing anthropogenic *p*CO_2_ alters seawater chemistry, with potentially severe consequences for coral reef growth and health. Octocorals are the second most important faunistic component in many reefs, often occupying 50% or more of the available substrate. Three species of octocorals from two families were studied in Eilat (Gulf of Aqaba), comprising the zooxanthellate *Ovabunda macrospiculata* and *Heteroxenia fuscescens* (family Xeniidae), and *Sarcophyton* sp. (family Alcyoniidae). They were maintained under normal (8.2) and reduced (7.6 and 7.3) pH conditions for up to 5 months. Their biolological features, including protein concentration, polyp weight, density of zooxanthellae, and their chlorophyll concentration per cell, as well as polyp pulsation rate, were examined under conditions more acidic than normal, in order to test the hypothesis that rising *p*CO_2_ would affect octocorals. The results indicate no statistically significant difference between the octocorals exposed to reduced pH values compared to the control. It is therefore suggested that the octocorals' tissue may act as a protective barrier against adverse pH conditions, thus maintaining them unharmed at high levels of *p*CO_2_.

## Introduction

The continuing rise in carbon dioxide emissions has already led to its increased concentration in the earth's atmosphere, from a level of 280 ppm in the pre-industrial era, to 385 ppm at present, which is the highest recorded in the last 650,000 years (Siegenthaler et al. 2005). On the basis of the emission trajectories, these levels are expected to continue to rise, reaching 800 ppm toward the end of the present century (Caldeira and Wickett 2005). Approximately one quarter of all anthropogenic carbon dioxide is currently absorbed by the oceans, causing changes in seawater chemistry by producing hydrogen protons, thus increasing seawater acidity and lowering the pH (e.g., Kleypas and Langdon 2006). This effect has already caused a drop in the average ocean surface water pH by 0.1 units since the pre-industrial era (e.g., Raven et al. 2005; IPCC, Climate Change 2007), and it is expected to decrease a further 0.4 pH units by the end of this century (e.g., Caldeira and Wickett 2003; Orr et al. 2005; Beman et al. 2011).

Numerous calcifying marine organisms produce a certain type of external organic layer, which can function as a physical barrier, separating their internal environment from the ambient seawater. For example, stony corals precipitate their aragonite skeleton beneath epithelial tissues (Allemand et al. 2004), crustaceans enclose their carapace within a relatively thick organic epicuticle (Ries 2011), and mollusks possess an external organic periostracum (Ries et al. 2009; Rodolfo-Metalpa et al. 2011). Certain biological features determine the degree to which organisms can tolerate changes in seawater pH. For example, low metabolic rate and life under little natural variation in carbon dioxide appear to characterize the more sensitive taxa to lower pH (e.g., Seibel and Walsh 2003; Pane and Barry 2007; Fabry et al. 2008). In the long-term, decreased seawater pH can lead to acidosis of the organism, and thus indirectly affect its growth (Marubini et al. 2008). Experimental study has suggested that such changes may have a profound impact on calcifying marine biota, such as coralline algae, foraminifers, reef-building corals, mollusks, echinoderms, etc., which rely on the delicate balance of dissolved inorganic carbon in the ambient seawater (e.g., Albright et al. 2008; Jokiel et al. 2008; Kroeker et al. 2010). Fine and Tchernov (2007) demonstrated that two Mediterranean stony coral species, maintained in highly acidified water, lost their skeleton, but regrew it after being returned to normal pH conditions. Kurihara and Shirayama (2004) reported for sea urchins, a reduced fertilization success, as well as reduced developmental rate and skeletogenesis with increasing CO_2_. In contrast, other studies have revealed that some organisms may exhibit enhanced calcification rate at high CO_2_ levels (e.g., Langer et al. 2006; Ries et al. 2009; Kroeker et al. 2011). It seems, therefore, that the response to ocean acidification is more complex than initially considered and may vary among taxa (Doney et al. 2008).

**Figure 1:**
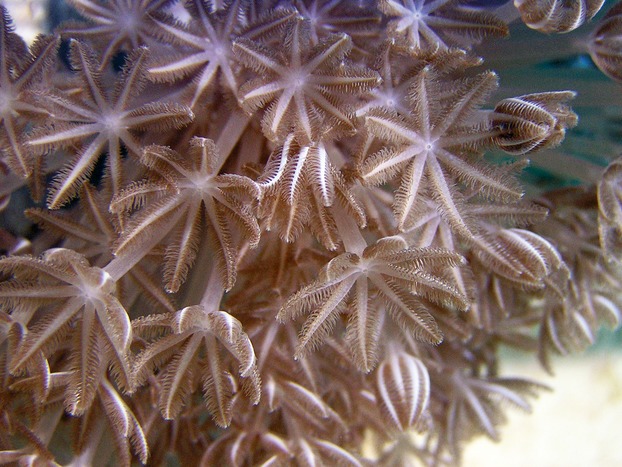
Polyps of the octocoral *Heteroxenia fuscescens*. Photo: Yasmin Gabay.

To date, no study has dealt with the possible effects of decreased seawater pH on octocorals, a dominant benthic component of many coral reefs (e.g., Fabricius and Alderslade 2001; Tentori and Allemande 2006). Octocorals feature an internal calcium carbonate skeleton comprised of microscopic sclerites embedded in the tissue (Fabricius and Alderslade 2001; Jeng et al. 2011; Tentori and Ofwegen 2011). The current study examined the effects of declining seawater pH on certain biological features of reef-dwelling octocorals at Eilat (northern Gulf of Aqaba, Red Sea). It encompassed three common Red Sea species: the zooxanthellate *Ovabunda macrospiculata* and *Heteroxenia fuscescens* (Fig. 1) (family Xeniidae), and *Sarcophyton* sp. (family Alcyoniidae). We tested the hypothesis that their biological features would be affected by the declining pH, and studied its effects on: (1) zooxanthellae density of *O. macrospiculata* (colonies), *H. fuscescens* (colonies and primary polyps), and *Sarcophyton* sp.; (2) chlorophyll concentration; (3) ratio of sclerite weight to tissue weight in *O. macrospiculata*; and (4) pulsation rate of *O. macrospiculata* polyps. As octocorals constitute an ecologically conspicuous benthic component on coral reefs (e.g., Benayahu and Loya 1981; Jeng et al. 2011), it is important to predict their response to a scenario of increased *p*CO_2_, thereby indicating their possible fate if such an environmental stressor will prevail.

## Materials and Methods

### Animals and experimental system

The study was conducted at the Interuniversity Institute for Marine Sciences in Eilat (IUI) (29°30′N 34°55′E), and the octocoral colonies were collected by SCUBA (2009–2010) from the reef adjacent to the IUI at a depth of 8-12 m. Following 2 weeks acclimatization in a flow-through seawater table, the colonies were transferred to the experimental system (see ahead). To obtain *H. fuscescens* planulae, colonies were collected and transferred into aquaria with running seawater (Yacobovitch et al. 2003). The following morning released planulae were pipetted out and transferred to Petri dishes, with preconditioned microscope slides or water papers (2 weeks immersion on the reef) as settlement substrate, for 2–3 weeks. After the planulae had metamorphosed into primary polyps, they were transferred to the experimental pH system. The system consisted of three water tables with two pH treatments: pH 7.6 and 7.3 (*p*CO_2_ = 1917 and 3898 *μ*atm, respectively), and a control pH 8.2 (*p*CO_2_ = 387 *μ*atm), corresponding to the ambient Eilat seawater (Silverman et al. 2009). Table 1 presents the seawater chemistry. The treatment values were determined following preliminary experiments that had revealed only a minor response of the octocorals to pH 7.9. We therefore selected a lower pH of 7.3, which is twice as high, in terms of *p*CO_2_, as that needed to reach pH 7.9. The diurnal pH fluctuations in Eilat reefs are in the order of 0.1 units, similar to that recorded in the pH system used in the current study.

**Table 1:** Carbonate chemistry parameters of treatments and control calculated from pH, total alkalinity, temperature (25°C), and salinity (40 ppm) using the program CO2SYS (Lewis and Wallace, 1998)

pH NBS	TA (μeqv kg^−1^)	DIC (μmol kg^−1^)	*p*CO_2_ (μatm)	CO_2(aq)_ (μmol kg^−1^)	HCO_3_^−^ (μmol kg^−1^)	CO_3_^2−^ (μmol kg^−1^)	Ω_arg_
8.2	2501	2122	387	10.6	1846	265	4.02
7.6	2499	2431	1917	52	2295	82	1.25
7.3	2501	2544	3898	107.1	2393	44	0.67

The selected species, *O. macrospiculata* (*n* = 100 colonies), *H. fuscescens* (*n* *=* 15 adult and 70 primary polyps), and *Sarcophyton* sp. (twenty-one 3 × 3 cm fragments), were equally divided among the two pH treatments (7.3 and 7.6) and the control (8.2). The colonies and primary polyps were placed in transparent 6 L PVC containers, supplied with an air stone (JUN ACO-5503, China Air pump; Guangdong Hailea Group Co., Ltd., Raoping County, Guangdong Province, China), and positioned under 400 W metal halide lamps, supplying ∼200 *μ*mol quanta m^−1^s^−1^, 10 h light: 14 h dark regime.

For the experimental system, seawater was pumped from a depth of 30 m at the IUI reef into 1000 L tanks, where the pH was regulated. The pH values (i.e., 7.3, 7.6, and 8.2) were achieved by bubbling pure CO_2_ gas stored in a cylinder through seawater to reach the desired pH. A pH electrode (S-200C; Sensorex, California) was located in each tank and connected to a pH controller (Aquastar; IKS ComputerSystem GmbH, Karlsbad, Germany) in order to control the gas flow. A pH deviation within the tank triggered the computer to activate the solenoid in order to either increase or decrease the flow of CO_2_, as necessary. The pH data were recorded using monitoring software (Timo, Matuta, Germany). The seawater temperature was maintained at ∼25°C, using a combination of an array of 150W BluClima aquarium heaters (Ferplast Spa, Vicenza, Italy) and an air-conditioner in the laboratory. Water flow in the tanks was maintained by power heads (At-301; Atman, Xiaolan Town, Zhongshan City, Guangdong Province China).

Three experiments were conducted for *O. macrospiculata* (April–May 2009, February–May 2010, and August–September 2010; in the last one only pulsation was measured), one for *H. fuscescens* (colonies: February–May 2010, primary polyps: October–November 2009), and one for *Sarcophyton* sp. (June–October, 2008). After polyps were removed from the colonies of *O. macrospiculata* in order to determine the biological features*,* the colonies were returned to the IUI reef, whereas those of *H. fuscescens* were repeatedly sampled. Primary polyps of *H. fuscescens* and *Sarcophyton* sp. fragments were sacrificed for the measurements. The octocorals were deliberately deprived of food.

### Biological assays

In order to test the effect of the pH treatments on the biological features of the octocorals, experiments were conducted over a period of 30–90 days in Xeniidae and 5 months in *Sarcophyton* sp. At different time points (see Results), samples comprised of six randomly selected polyps from each colony of *O. macrospiculata*, five from *H. fuscescens* (*n* = 2–8 colonies), and a fragment from *Sarcophyton* sp. (*n* = 5–7 fragments), were placed in filtered seawater (FSW, 0.2 *μ*m pore-size). Each sample was separately homogenized (Diax Heidolph Instruments, Germany), and its total volume was determined to a precision of 0.05 mL and centrifuged (Sigma 4k15; Sigma laborzentrifugen GmbH, Osterode, Germany) for 5 min at 5000 rpm and 4°C, in order to separate the algal cells from the tissue, where applicable. A sample of 100 *μ*L was removed from the supernatant for protein determination of the tissue using Quick Start Bradford Protein Assay Kit (Bio-Rad Laboratories, Hercules, CA). Optical density was read at 595 nm using an ELISA reader (PowerWave XS; BioTek, Winooski, Vermont), and the concentration was calculated according to the Quick Start Bovine Serum Albumin Standard Set (Bradford 1976). In order to obtain the total amount of protein (mg), the subsample protein concentration was multiplied by the total volume of each one (see above) and normalized to zooxanthellae count.

The supernatant of the samples was discarded and the pellet containing the dinoflagellate-cells was resuspended in 1 mL of FSW, homogenized and then centrifuged (for 5 min at 2795 g). Finally, clean dinoflagellate-cells were obtained and a sample of 50 *μ*L was removed for photographic count, using a digital camera (CoolPix 995; Nikon, Japan) attached to a microscope (Nikon Eclipse TE 2000-E; NIKON CORPORATION, Chiyoda-ku, Tokyo, Japan). The dinoflagellates were manually counted in the photos, using ImageJ © program (Cell Counter application), and multiplied by 10,000 to obtain their total amount in a field size of 0.1 × 0.1 cm surface × 0.01 cm depth. In order to obtain the chlorophyll concentration of the zooxanthellae*,* the remaining content of each sample was centrifuged again, 1 mL of cold acetone 90% (4°C) was added to the pellet, and it was incubated at 4°C for 18 h in the dark. Chlorophyll *a* concentration was determined using spectrophotometry (Ultrospec 2100 pro; GE Bioscience, Piscataway, New Jersey), following Jeffrey and Humphrey (1975).

An additional sample of six polyps of *O. macrospiculata* (tissue and sclerites) was dried overnight and then weighed using analytic balance (ViBRA AJ-320CE; Yushima, Bunkyo-ku, Tokyo, Japan; precision 10^−3^). Their sclerites were obtained by dissolving the tissue with 10% sodium hypochlorite, followed by repeated rinsing in double-distilled water (DDW), and then a wash with 95% alcohol (Aharonovich and Benayahu 2011). The alcohol was removed and the tubes were kept open overnight at room temperature to dry. The sclerites of each sample were weighed and the ratio between sclerite weight to polyp weight in *O. macroscpiculata* was determined.

The possible effect of decreased *p*CO_2_ on pulsation rate of *O. macrospiculata* polyps was examined in colonies maintained in the experimental system, by means of three video recordings taken from five colonies (1 min each; Canon PowerShot G9 camera, Ohta-ku, Tokyo, Japan). Six polyps were randomly chosen from each video per colony and the number of pulses of the polyps per 1 min was counted and averaged (±SD). In the reef, the pulsation of colonies was determined by underwater video recordings, using a Canon PowerShot G9 camera, Japan (December 2010, between 1000 and 1200 h). Each colony was photographed three times for 1 min (*n* = 5 colonies), and the number of pulses of six randomly chosen polyps per 1 min was calculated.

### Statistical analysis

Analysis of variance (ANOVA) was performed on the data using SPSS 15.0 (IBM Corporation, Armonk, New York) and STATISTICA 8 (StatSoft, Inc, Tulsa, Oklahoma). Log transformation was conducted on part of the data in order to achieve normal distribution (see Results). Results are expressed as mean ± standard deviation (SD).

## Results

Throughout the experiments, little mortality was noted among the octocoral colonies and primary polyps, and they maintained their normal appearance with no visible signs of stress. Data for pH 7.3 are not available for the second experiment, February–May 2010, on *O. macrospiculata* and *H. fuscescens*, due to a technical fault in the experimental system.

### Zooxanthellae density

**Figure 2:**
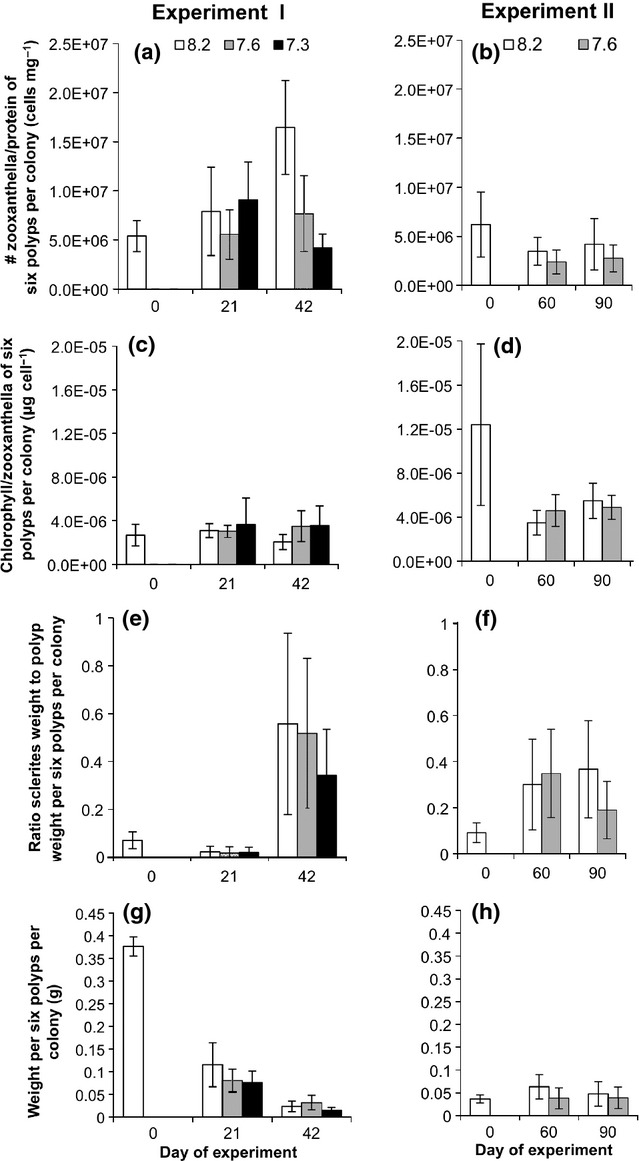
*Ovabunda macrospiculata*: Biological response under normal (pH 8.2) and experimental (pH 7.6, 7.3) conditions over time (±SD), in experiments I (April–May 2009) and II (February–May 2010). (a, b) Average number of zooxanthella-cells per mg tissue protein (*n* = 7–8 colonies, 6 polyps/colony); (c, d) Average *μ*g chlorophyll per zooxanthella-cell (*n* = 7–8 colonies, 6 polyps/colony; (e, f) Average ratio of sclerite weight to polyp weight per colony (*n* = 6–8 colonies, 6 polyps/colony; (g, h) Weight of 6 polyps/colony (*n* = 8 colonies/treatment). Data for pH 7.3 are not available for the second experiment (February–May 2010) due to a technical fault in the experimental system.

**Figure 3:**
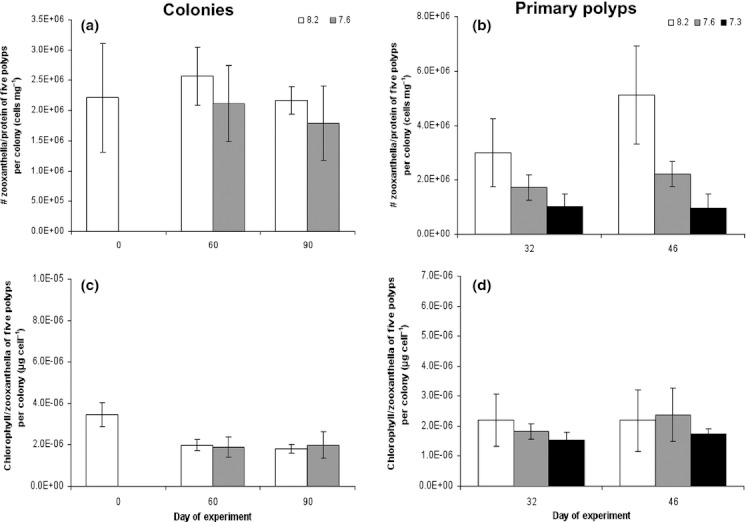
*Heteroxenia fuscescens*: Biological response under normal (pH 8.2) and experimental (pH 7.6, 7.3) conditions over time (±SD), in colonies and primary polyps. (a, b) Average number of zooxanthella-cells per tissue protein of five polyps per colony (cells mg^−1^); (c, d) Average chlorophyll content per zooxanthella-cell of 6 polyps/colony (*μ*g cell^−1^). *n* = 2–5 colonies/treatment, *n* = 3 primary polyps/treatment. Data for pH 7.3 are not available due to a technical fault in the experimental system.

**Figure 4:**
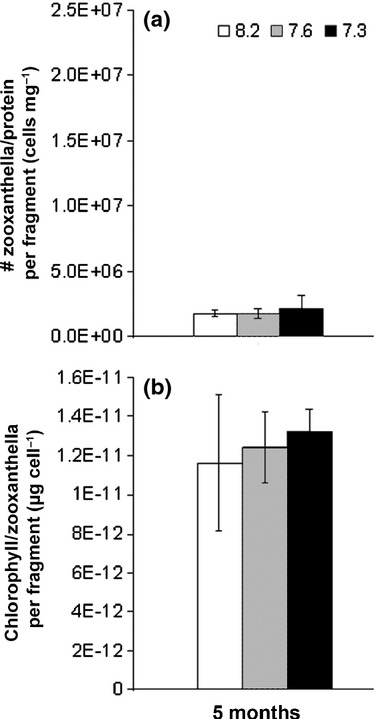
*Sarcophyton* sp.: Biological response under normal (pH 8.2) and experimental (pH 7.6, 7.3) conditions after a 5-month incubation period (±SD). (a) Average number of zooxanthella-cells per tissue protein (cells mg^−1^) (*n* = 5–7 fragments/treatment); (b) Average chlorophyll content per zooxanthella-cell (*μ*g cell^−1^). *n* = 6–7 fragments/treatment.

The average number of zooxanthella-cells per tissue protein (cells mg^−1^) in *O. macrospiculata* was not significantly affected over time by *p*CO_2_ in the two experiments, with no such differences between treatments and control on each of the sampling dates of both experiments (Fig. 2a and b, following log transformation, two-way ANOVA, *P* = 0.75, 0.08, respectively). The results of the first experiment (April–May 2009) ranged between 4.17^6^± 1.41^6^ (pH 7.6, day 42) and 1.64^7^± 4.77^6^ (pH 8.2, day 42) cells per mg protein; and of the second one (February–May 2010), between 2.38^6^± 1.23^6^ (pH 7.6, day 60) and 6.19^6^± 3.30^6^ (pH 8.2, day 0) cells mg^−1^. In colonies of *H. fuscescens* (February–May 2010), the average number of zooxanthella-cells per tissue protein was not significantly affected by *p*CO_2_ (Fig. 3a: repeated measures ANOVA, *P* = 0.06). The colonies featured 1.79^6^± 6.19^5^ (pH 7.6, day 90) to 2.57^6^± 4.83^5^ (pH 8.2, day 60) algal cells per mg protein. Similarly, the primary polyps of *H. fuscescens* in October–November 2009 were not affected over time by *p*CO_2_ (Fig. 3b: two-way ANOVA, *P* = 0.11) and the results ranged between 1.01^6^± 4.75^5^ (pH 7.3, day 32) and 2.99^6^± 1.24^6^ (pH 8.2, day 32) cells mg^−1^. The average number of zooxanthella-cells per tissue protein of *Sarcophyton* sp. (June–October, 2008) was also not significantly affected by *p*CO_2_ (Fig. 4a: following log transformation, one-way ANOVA, *P* = 0.59) and the results ranged between 1.77^6^± 2.51^5^ (pH 8.2) and 2.10^6^± 1.10^6^ (pH 7.3) cells mg^−1^.

### Chlorophyll concentration

The chlorophyll content per zooxanthella-cell (*μ*g cell^−1^) in *O. macrospiculata* was not significantly affected over time by *p*CO_2_ in either the April–May 2009 or February–May 2010 experiments, with no such differences between treatments and control on each of the sampling dates of both experiments (Fig. 2c and d: two-way ANOVA, *P* = 0.14, 0.82, respectively). The results of the first experiment (April–May 2009) ranged between 2.05^−6^ ± 6.89^−7^ (pH 8.2, day 42) and 3.64^−6^ ± 2.44^−6^ (pH 7.3, day 42) *μ*g chlorophyll per algal cell; and that of the second (February–May 2010) between 1.24^−5^ ± 7.34^−6^ (pH 7.6, day 60) and 5.46^−6^ ± 1.61^−6^ (pH 8.2, day 90) *μ*g cell^−1^. Similar results were obtained for colonies of *H. fuscescens* (February–May 2010) (Fig. 3c: repeated measures ANOVA, *P* = 0.15). The primary polyps of a *H. fuscescens* (October–November 2009) were also significantly unaffected by *p*CO_2_ (Fig. 3d: two-way ANOVA, *P* = 0.17), for which the results ranged between 1.53^−6^ ± 2.49^−7^ (pH 7.3, day 32) and 2.37^−6^ ± 8.91^−7^ *μ*g cell^−1^ (pH 7.6, day 46). The average chlorophyll content per zooxanthella-cell (*μ*g cell^−1^) of *Sarcophyton* sp. (June–October, 2008) was also not significantly affected by *p*CO_2_ (Fig. 4b: one-way ANOVA, *P* = 0.18), and the results ranged between 1.16^−5^ ± 3.50^−6^ (pH 8.2) and 1.32^−5^ ± 1.16^−6^ (pH 7.3) *μ*g cell^−1^.

### Ratio of sclerite weight to polyp weight

Average ratio between sclerite weight and polyp weight (tissue and sclerites) of *O. macrospiculata* did not significantly differ between the pH treatments and the control for both the 2009 and 2010 experiments (Fig. 2e and f: following log transformation, two-way ANOVA, *P* = 0.50, 0.72, respectively). The results of the first experiment (April–May 2009) ranged between 0.018 ± 0.026 (pH 7.6, day 21) and 0.557 ± 0.378 (pH 8.2, day 42); and of the second one (February–May 2010) between 0.092 ± 0.043 (pH 8.2, day 0) and 0.367 ± 0.211 (pH 8.2, day 90).

Similarly, the average weight of polyps per colony of *O. macrospiculata* did not indicate significant differences between pH treatments and control in both the 2009 and 2010 experiments (Fig. 2g and h: two-way ANOVA, *P* = 0.38, 0.33 respectively).

### Pulsation rate

**Figure 5:**
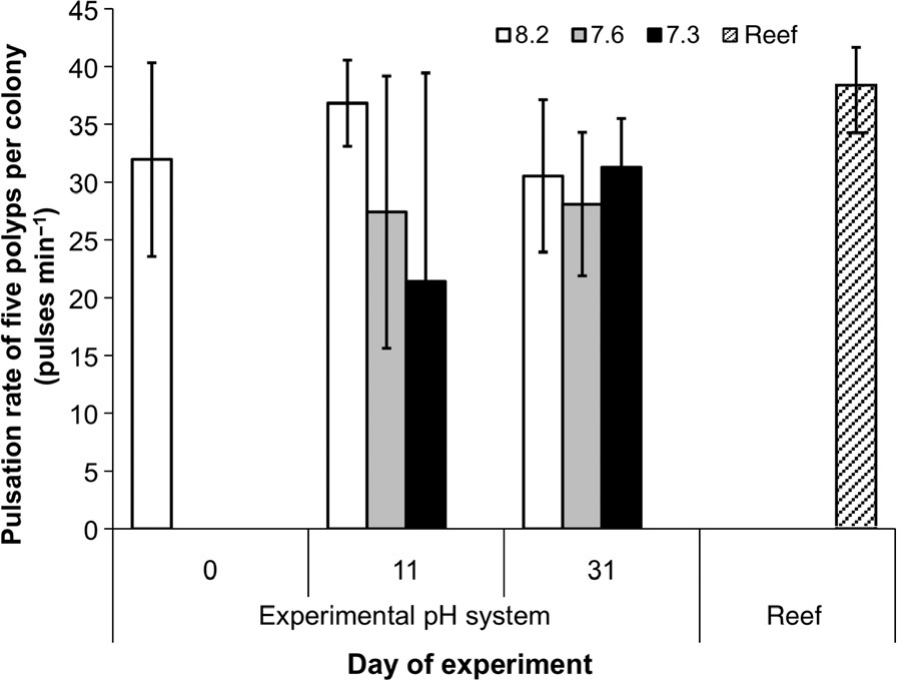
*Ovabunda macrospiculata*: Pulsation rate of five polyps per colony (pulses min^−1^) under normal (8.2) and reduced (7.6 and 7.3) pH conditions (±SD) over time, *n* = 2–6 colonies/treatment.

The pulsation rate of *O. macrospiculata* was not significantly affected over time by *p*CO_2_, with no such differences between treatments and control on each of the measurement dates (Fig. 5: two-way ANOVA, *P* = 0.32). Similarly, there was no significant difference between the pulsation rate of colonies in the reef and in the experimental pH system (two-way ANOVA, *p*_reef_ = 0.18). The results ranged between 21.39 ± 18.06 (pH 7.3, day 11) and up to 36.83 ± 3.73 pulses min^−1^ (pH 8.2, day 11), with a pulsation rate of reef colonies averaging 38.20 ± 3.48 pulses min^−1^.

## Discussion

The current study examines for the first time the biological features of zooxanthellate reef octocorals, whose biological features were examined under high levels of *p*CO_2._ The results reveal that the octocorals remained statistically unaffected under such conditions over a period of up to 5 months. The number of zooxanthellae per tissue protein in colonies of *O. macrospiculata* (Fig. 2a and b), in colonies and primary polyps of *H. fuscescens* (Fig. 3a and b), and in fragments of *Sarcophyton* sp. (Fig. 4a and b), did not significantly differ between pH treatments and control. Crawley et al. (2010) reported no changes in zooxanthellae per surface area of the stony coral *Acropora formosa* (Orpheus Island, Australia) following exposure to lower pH. However, other studies found contradicting patterns of change, such as Krief et al. (2010), who found in the two Red Sea stony corals *Porites* sp. and *S. pistillata*, a decrease in zooxanthellae density per tissue protein as pH decreased (∼25). Similarly, Anthony et al. (2008) found 40–50% loss of zooxanthellae in *A. intermedia* (Heron Island), whereas Reynaud et al. (2003) reported an increase in algal density per host cell with decreased pH in *S. pistillata* (at ∼25°C). Overall, this suggests species-specific responses, but also that different experimental conditions, such as light intensity and temperature, may yield different patterns of biological responses to increased *p*CO_2_ (e.g. Reynaud et al. 2003 cf. Krief et al. 2010).

The present study demonstrates that chlorophyll level per zooxanthella-cell was not significantly affected by *p*CO_2_ in colonies of *O. macrospiculata* (Fig. 2c and d), colonies and primary polyps of *H. fuscescens* (Fig. 3c and d), and fragments of *Sarcophyton* sp. (Fig. 4a and b). Reynaud et al. (2003) and Marubini et al. (2008) obtained similar results under high *p*CO_2_ levels for *S. pistillata*. However, both Anthony et al. (2008) and Krief et al. (2010) demonstrated for *A. intermedia*, *Porites* sp., and *S. pistillata,* an increased chlorophyll concentration per algal-cell at higher *p*CO_2_ levels (up to pH 7.19), which they explained as compensation for the recorded decrease in algal cells. Crawley et al. (2010) reported an increase in chlorophyll concentration in *A. formosa* (Orpheus Island) with no change in zooxanthellae density, even at pH 7.55, probably due to the short exposure time (4 days) and low light (110 *μ*mol m^−1^ s^−1^). Photo-acclimation is a dynamic and immediate process reflected in rapid changes in chlorophyll within 2–4 days, whereas changes in zooxanthellae density may occur over a longer period of up to 40 days (Titlyanov et al. 2001). In this study the octocorals were exposed to high *p*CO_2_ conditions for up to 5 months, which can be considered long enough to cause changes in chlorophyll and zooxanthellae density; and yet no significant changes were noted. Therefore, it is suggested that the symbiotic relationship between these octocoral hosts and their algal symbionts, as well as the photosynthetic activity of the zooxanthelae, were not significantly affected under the experimental conditions.

The ratio of sclerite weight to polyp weight of *O. macrospiculata* was not significantly affected by increasing *p*CO_2_ (Fig. 2e–h). These findings stand in contrast with the majority of studies performed with scleractinian corals, which have revealed a decreased skeleton growth of up to 40% with increased *p*CO_2_ (e.g., Langdon and Atkinson 2005; Schneider and Erez 2006; Fine and Tchernov 2007; Anthony et al. 2008; Krief et al. 2010). Using buoyant weight, Rodolfo-Metalpa et al. (2010) found that colonies of the bryozoan *Myriapora truncate* maintained their calcification rate even under such low pH conditions as 7.66. Similarly, Moy et al. (2009) showed a 30–35% reduction in shell weight in the foraminifer *Globigerina bulloides* that was consistent with the decrease in calcification rate in stony corals. Our own results revealed that the ratio between sclerite weight and polyp weight or protein content was not significantly affected by the decreasing ambient pH.

Polyp pulsation, a unique phenomenon among xeniid octocorals (Reinicke 1997) including *O. macrospiculata*, is known to be sensitive to stressors, such as crude oil (Cohen et al. 1977). In the current study, the pulsation rate of its polyps was not significantly affected by the declining pH (Fig. 5). Using a closed system, Sprung and Delbeek (1997) noted that under pH 8.1, *Xenia* species lost their pumping (pulsating) coordination, and even the tentacular pinnules degenerated. In this study, variation in pulsation between colonies was demonstrated, but no such difference was found between pH treatments and control. Moreover, no difference was found between the system and the reef, which might indicate that the system itself does not exert additional pressure on the corals in term of pulsation. *O. macrospiculata* is a passive suspension feeder (Shimeta and Jumars 1991) and its pulsation may create a flow over the polyps. Although the precise biological implication of pulsation behavior remains to be studied, our results indicate that it is not significantly affected by declining pH, despite some variation being found among individuals.

Numerous experimental studies have been performed on the possible effects of ocean acidification on marine biota, including the calcification response of stony corals and coccolithophores (e.g., Kleypas et al. 2006). The present study is the first to examine the possible effects of rising *p*CO_2_ on octocorals. Although colonies of *O. macrospiculata*, *Sarcophyton* sp., and colonies and primary polyps of *H. fuscescens* were exposed to conditions more acidic than normal, their biological features were not significantly affected by the elevated *p*CO_2_. These findings indicate that octocorals may possess certain protective mechanisms against rising levels of *p*CO_2_. It is suggested that their fleshy tissues act as a barrier, maintaining a stable internal environment and avoiding the adverse effects of the ambient elevated *p*CO_2_ (Rodolfo-Metalpa et al. 2011). This suggestion is further supported by our finding that the ultrastructural features of *O. macroscipulata* sclerites are not affected by increased ambient seawater acidity (Y. Benayahu, M. Fine, and Y. Gabay unpubl. ms.) Most experimental studies on the effect of ocean acidification on marine organisms have only lasted from a few hours to several days, whereas the present study was run for 5 months. This further strengthens our suggestion that octocorals might be able to acclimate and withstand rising levels of ocean acidification, even under conditions that are far beyond what is expected to occur by the end of the present century (pH 7.9, see IPCC). The variable responses among taxa as a reaction to elevated *p*CO_2_ reflect differences in their ability to regulate their internal pH, and the extent to which their tissues can avoid changes in their response, including their mineral composition (Ries et al. 2009; Kroeker et al. 2010). Additional studies on the examined octocoral species are expected to strengthen the results and verify them. Studies on the mechanisms that may regulate the internal pH in octocorals, along with the effects of long-term exposure to ocean acidification conditions, are still needed in order to acquire a better understanding of the effects of elevated *p*CO_2_ on octocorals.
